# Role of less commonly agreed risk factors on disease recurrence in endometrial cancer: a propensity scorematched comparison

**DOI:** 10.4274/tjod.galenos.2019.24571

**Published:** 2019-03-27

**Authors:** Hülya Ayık Aydın, Gülgün Erdoğan, Hatice Elif Pestereli, Tayup Şimşek

**Affiliations:** 1 Akdeniz University Faculty of Medicine, Department of Obstetrics and Gynecology, Division of Gynecological Oncology, Antalya, Turkey; 2 Akdeniz University Faculty of Medicine, Department of Medical Pathology, Division of Gynecopathology, Antalya, Turkey

**Keywords:** Endometrial cancer, recurrence, risk factor, CA-125

## Abstract

**Objective:**

To compare the clinicopathologic features of patients with endometrial cancer (EC) with recurrent disease with a primary surgery, stage, grade, and tumor histotype-matched cohort of patients without recurrence.

**Materials and Methods:**

Patients with EC who were surgically treated at a single tertiary care institution between 2005 and 2015 were enrolled in this study. The dataset included 381 consecutive patients with EC, of which 31 (8.1%) had disease recurrence. Data consisting of age at surgery, CA- 125 concentration at diagnosis, number of lymph nodes harvested, growth pattern of the primary tumor, location of the primary tumor within the endometrium, tumor histotype, tumor grade, disease stage, adjuvant therapy, disease recurrence, time to recurrence, CA-125 concentration at recurrence, clinical and imaging findings at recurrence, and treatment modalities used for recurrent disease were extracted from the institutional database.

**Results:**

After 1-to-1 propensity-score matching of patients with and without recurrence, the clinicopathologic features of 26 patients from each group were compared. Patients with recurrent disease were found to have a significantly higher CA-125 concentration at initial diagnosis (p<0.001) and different tumor growth pattern (p=0.019) than patients without disease recurrence. The papillary growth pattern of the primary tumor was significantly associated with disease recurrence as compared with polypoid and infiltrative patterns. Omental involvement, papillary tumor growth, and advanced age were associated with increased mortality.

**Conclusion:**

Our results indicated that higher CA-125 concentrations at initial diagnosis and papillary growth pattern of primary tumors were found to be associated with disease recurrence.

**PRECIS:** Role of less commonly agreed risk factors on disease recurrence in endometrial cancer: A propensity score- matched comparison.

## Introduction

Endometrial cancer (EC) is the most common cancer of the female genitourinary system in the United States of America and the 4^th^ most common tumor in Western countries, with more than 280.000 patients appearing yearly, globally^(1)^. Nevertheless, data reveal that the mortality rate is rising more quickly than the incidence, possibly because of an improved rate of advanced-stage tumors, high-risk histopathologies, and cases being identified in the elderly^(2)^. Consequently, 13-17% of patients will develop disease recurrence, usually within three years of initial treatment^(3,4)^.

Although most with EC are diagnosed with early-stage disease and have a favorable prognosis, approximately 15% of these cases relapse^(5)^. Treatment for recurrence includes local treatment (radiotherapy or surgery), systemic chemotherapy or relevant combinations. It differs according to the involved site, recurrent disease extent, and types of previous therapy. The 3-year survival rate in patients with EC recurrence is reported to be between 8% and 73%^(6)^.This broad range indicates that patients with EC recurrence represent a heterogeneous group with different prognostic factors for survival. Thus, there is a need to better discriminate these patients depending on prognosis after relapse. Numerous features have been examined to evaluate their effect on recurrence; patient’s age at diagnosis, the stage, histologic type, cell type, cervical involvement, depth of myometrial invasion, and lymph node metastasis at the time of treatment were identified as the most significant prognostic factors in patients with EC^(7,8)^. Nevertheless, these factors are not satisfactory to precisely predict the prognosis of these patients.

There is a common consensus that disease stage, tumor histotype, and tumor grade are significantly associated with risk of recurrence in EC. To examine the roles of less commonly agreed risk factors on disease recurrence, we compared the clinicopathologic features of patients with recurrent disease with a primary surgery, stage, grade,and tumor histotype-matched cohort of patients without recurrence.

## Materials and Methods

### Study design

The study was approved by the Akdeniz University Faculty of Medicine Ethics Committee (no: 15/03.01.2018). All patients gave written informed consent, which allowed the participating institution to use their clinical data. Patients with EC who were surgically treated at a single tertiary care institution between 2005 and 2015 were enrolled in this study.

In the present study, recurrence was defined as any documented reappearance of cancer, either locally or systemically, after a disease-free interval of ≥3 months. Local-regional recurrence was defined as relapse at the vaginal vault or in the regional lymph nodes including pelvic and paraaortic nodes. Abdominal/peritoneal recurrence was defined as relapse at the peritoneum, omentum or serosal surfaces of the abdominal viscera, or occurrence of malignant ascites. Distant metastasis was defined as any relapse at extraabdominal organs and lymph nodes, or in the parenchyma of the intraperitoneal organs.

Routine post-remission surveillance at our institution was to follow up patients every three months for two years, every six months for the next three years, and then annually. At follow-up visits, a physical examination, ultrasonography, complete blood count, and blood chemistry including serum CA-125 level were performed. Thoracic and abdominal computed tomography scans were used six-monthly for two years and then annually. In the event of equivocal clinical or radiologic findings, positron emission tomography combined with computed tomography (PET-CT) was performed. Image-guided biopsy was used whenever possible, and there was a high suspicion of disease recurrence before the treatment of recurrence was started.

### Outcome parameters

Data consisting of age at surgery, CA-125 concentration at diagnosis, time from diagnosis to primary treatment, type of surgical procedure performed, number of lymph nodes harvested, growth pattern of the primary tumor, location of the primary tumor within the endometrium, tumor histotype, tumor grade, disease stage, adjuvant therapy, disease recurrence, time to recurrence, CA-125 concentration at recurrence, clinical and imaging findings at recurrence, largest recurrent tumor size, extent of recurrent disease, number of recurrent lesions, and treatment modalities used for recurrent disease were extracted from the institutional database.

### Statistical Analysis

The dataset included 381 consecutive patients with EC, of which 31 (8.1%) had disease recurrence. A 1-to-1 propensity score-matched analysis was conducted to provide matched cohorts of patients with and without recurrence, and thus, to reduce the impact of covariate bias. Multivariate logistic regression models were used to develop separate propensity scores. Disease stage, tumor grade, tumor histotype, and primary surgery [total hysterectomy/bilateral salpingo-oophorectomy (TH/BSO) alone vs. TH/BSO plus lymphadenectomy] were selected as potential confounding covariates because they are known to be independent factors for risk of recurrence and to be the main determinants of prognosis in patients with EC. For tight matching, nearest-neighbor matching was performed with a caliper width (the match tolerance) equal to 0.01, which resulted in successful matching of 26 patients from each group. A simple logistic regression analysis was then performed using the matched groups to assess independent associations of clinicopathologic factors and disease recurrence. The differences between two groups were tested using the Mann-Whitney U test for nonparametric data. The chi-square or Fisher’s exact tests were used for the comparison of categorical variables.

Statistical analyses were performed using NCSS (Number Cruncher Statistical System) 2007 statistical software (NCSS LLC, Kaysville, Utah, USA). A two-tailed p<0.05 was considered statistically significant.

The cut-off point for CA-125 was determined using receiver operating characteristics (ROC) analysis based on the survival data. Survival analysis was performed to investigate the survival probabilities of variables. The mean durations of survival, 95% confidence intervals, as well as hazard ratios are presented.

## Results

Table 1 shows the baseline characteristics of 381 patients with EC. Eighty percent of patients had endometrioid tumor histotype. According to the International Federation of Obstetricians and Gynecologists (FIGO) grading system, 52% of patients had grade 1 tumor, 30.2% had grade 2, and 17.6% had grade 3. The patients treated with TH/BSO plus lymphadenectomy accounted for 90.6%. The median number of lymph nodes harvested was 30. The FIGO_2009_ stages of the patients were as follows: stage IA, 179 patients (47.0%); stage IB, 110 patients (28.9%); stage II, 25 patients (6.6%); stage IIIA, 8 patients (2.1%); stage IIIB, 1 patient (0.3%); stage IIIC, 46 patients (12.1%); and stage IVB, 12 patients (3.1%). Adjuvant therapy was given to 55.4% of the patients. Thirty-one patients (8.1%) developed disease recurrence during a median follow-up time of 68 months.

The characteristics of the 31 patients with recurrent disease are presented in Table 2. The median time to recurrence was 15 (range, 3 to 46) months. Only five patients (16.1%) first presented with abnormal clinical signs and/or symptoms, but none had positive physical findings alone. At the time of the diagnosis of recurrence, all patients had positive imaging findings and 26 (83.9%) had elevated CA-125 concentrations. On the other hand, only 2 (6.5%) patients were incidentally diagnosed throuhg routine imaging studies alone. The majority (74.2%) of patients developed multiple or disseminated metastases. Recurrent disease was limited to the vaginal vault and/or regional lymph nodes in only 19.4% of patients. The liver was the most common site of distant metastasis, found in 22.6% of patients.

After 1-to-1 propensity-score matching of patients with and without recurrence based on stage, grade, histotype, and primary surgery, the clinicopathologic features of 26 patients from each group were compared (Table 3). Study groups were comparable for age at surgery, time from diagnosis to primary treatment, number of lymph nodes harvested, location of the primary tumor within the endometrium, lymphovascular space involvement, cervical invasion, positive peritoneal cytology, and adjuvant therapies received. However, patients with recurrent disease were found to have a significantly greater tumor size (p=0.026), higher CA-125 concentration at initial diagnosis (p<0.001), and different tumor growth pattern (p=0.019) than patients without disease recurrence. The papillary growth pattern of the primary tumor was significantly associated with disease recurrence as compared with polypoid and infiltrative patterns.

Elevated CA-125 concentration and papillary growth pattern did not exhibit a significant difference between type I and type II cancers (p=0.267 and p=0.429, respecetively). Table 4 demonstrates a comparative overview for histological grade, histological type, and staging data for tumors with and without recurrence. As would be expected, advanced grade and tumor stage were significantly associated with increased recurrence.

As shown in Table 5, analysis of the impacts of parameters on survival indicated that patients with tumoral involvement of omentum were at 5.712-times greater risk for mortality. Papillary tumors were 4.67 times more lethal compared with non-papillary neoplasms. Mortality was 1.041 times more likely for every advanced year.

**Figure 1:**
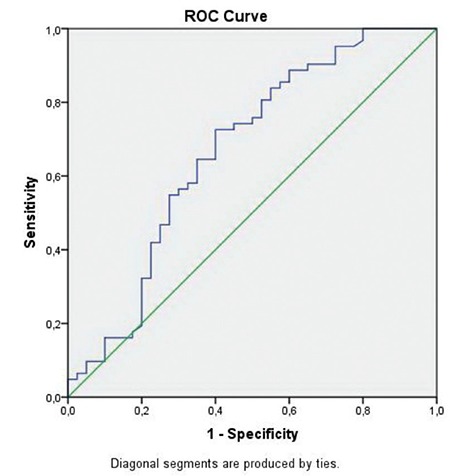
ROC curve for CA-125 levels

ROC analysis was used to determine the cut-off point for CA-125 concentrations. Taking mortality into account as the gold standard, the cut-off point was determined as 44.1 with a sensitivity and specificity of 72.6% and 60%, respectively. The ROC curve is demonstrated in Figure 1.

**Figure 2:**
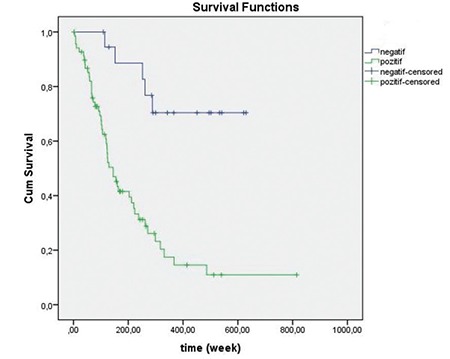
Kaplan-Meier analysis for omental involvement

**Figure 3:**
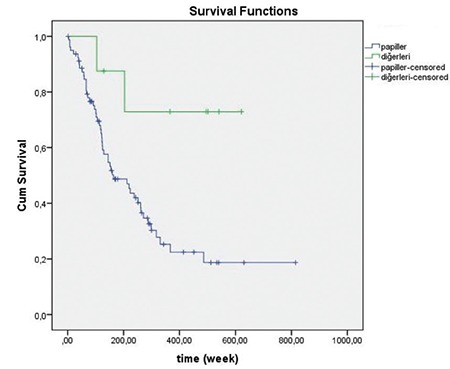
Kaplan-Meier analysis for papillary tumor growth pattern

Kaplan-Meier analyses for omental involvement and papillary tumor patterns are indicated in Figures 2 and 3, respectively.

## Discussion

In the present study, we aimed to compare the clinicopathologic features of patients with recurrent disease with a primary surgery, stage, grade, and tumor histotype-matched cohort of patients without recurrence. We found that higher CA-125 concentrations at initial diagnosis and a papillary growth pattern of the primary tumor were associated with disease recurrence.

In the present study, the ratio of recurrence after the initial treatment of EC was 8.1%. This rate is slightly lower than previously described in other reports^(4,5,6)^. The low recurrence rates obtained in our study may be due to the patient characteristics in the study group or may be a result of the extensive surgical interventions and/or adjuvant radiotherapy, and chemotherapy being applied. The relation of EC recurrences and the interval passed from initial treatment for EC indicates that time to recurrence was detected with a median of 15 months. These data are in accordance with ratios described by other reports^(6,7,8)^. 

In the present study, our data confirmed a tendency described by the mainstream of reports that distant recurrences are more frequent than local recurrences after the initial treatment of EC. This is possibly due to pelvic adjuvant radiotherapy following surgical procedures. The present study demonstrated findings that are very comparable to the other reports^(9,10)^. Sohaib et al.^(9)^ reported 34.4% local recurrences, 46.9% distant recurrences, and 18.8% in both sites, and Fung‑Fee‑Fung et al.^(10)^ reported 61% of distant metastases including multifocal relapses and 39% of local recurrences.

Although serum concentrations of CA-125 have been used as a worthy tumor marker for EC diagnosis and recurrence detection after the initial treatment, their role as a useful tumor marker in EC is the subject of debate^(11)^. Several reports have revealed that serum levels of CA-125 are critical for preoperative diagnosis and prediction of the recurrence, and that their elevation was associated with advanced-stage EC^(12)^. Yildiz et al.^(13) ^showed that the CA-125 serum concentration increase was related with the upsurge in extra-uterine disease incidence. Similarly, Chen et al.^(14)^ revealed that elevated CA-125 concentrations were intensely correlated with lymph node metastasis, advanced surgical stage, and poor prognosis^(13,14)^. Elevated serum concentrations of CA-125 at initial diagnosis are linked with extrauterine tumor extension and lymph node metastases^(15,16)^. In the present study, patients with recurrent disease were observed to have a significantly higher CA-125 concentration at initial diagnosis.

Abnormal preoperative cytology was demonstrated to be associated with a number of poor prognostic features such as tumor grade, degree of myometrial invasion, and lymph node spread^(17,18,19)^. Abnormal cytology is correlated with advanced-stage for both endometrioid and papillary serous or clear cell histologic subtypes^(18,19)^. The aggressive performance of the papillary subtype was firstly defined by Hendrickson et al.^(20)^ as a subtype of EC with precise pathologic and clinical features related with a high frequency of tumor recurrence. Papillary subtype represents 3-10% of all ECs but accounts for nearly 40% of EC deaths^(20,21)^. In a recent study of patients with EC, cervical cytology was revealed to be an individual predictor of lymph node spread^(18)^. Regardless of the strong connection between abnormal cytology and recurrence, the direct association between abnormal cytology and recurrence risk has not been comprehensively assessed. Fukuda et al.^(19)^ stated that abnormal cytology was associated with disease-free survival in univariate analysis but was not an independent predictor of survival on multivariate analysis. In the present study, papillary growth pattern of the primary tumor was significantly associated with disease recurrence as compared with polypoid and infiltrative patterns. Our results yielded that omentum involvement, papillary-type tumor growth, and advanced age were linked with mortality risk.

There is an increasing awareness in observational reports of the need to assess the effects of treatment on results. In observational and nonrandomized studies, treatment choice is frequently affected by patient individualities. Consequently, researchers have to account for for systematic alterations in baseline features amongst compared groups when assessing the result of treatment on outcomes. Lately, when using observational data, there has been growing concern regarding techniques established on the propensity score to decrease or eradicate the outcomes of confounding^(22)^.

Some limitations of the present study should be mentioned. First, the retrospective nature of the study causes bias. Secondly, this was a single-institution study, and caution must be taken before generalizing the conclusions to further settings. Another limitation is the long study period because study group comprised patients who experienced distinct surgical treatment (i.e*.* systematic pelvic lymphadenectomy was only recommended for high-risk ECs from 2010)^(23)^. Thirdly, physical activity, diet, and presence of chronic diseases such as hypertension or diabetes were not taken into account, though numerous investigators have formerly shown that these features might normalize endometrial hormone-receptor expressions and definitely effect survival^(24,25)^.

## Conclusion

Higher CA-125 concentrations at initial diagnosis and a papillary growth pattern of the primary tumor were found to be associated with disease recurrence. Omental involvement, papillary tumor growth, and advanced age were found to increase mortality rates. Further randomized, prospective, controlled trials on larger series are necessary for making more precise interpretations.

## Figures and Tables

**Table 1:**
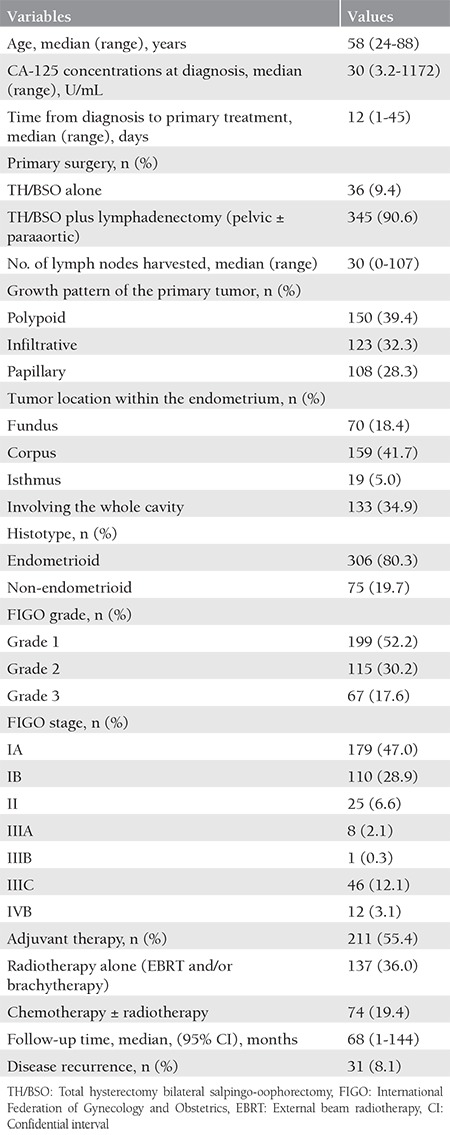
Initial surgical and pathologic characteristics of 381 patients with endometrial cancer

**Table 2:**
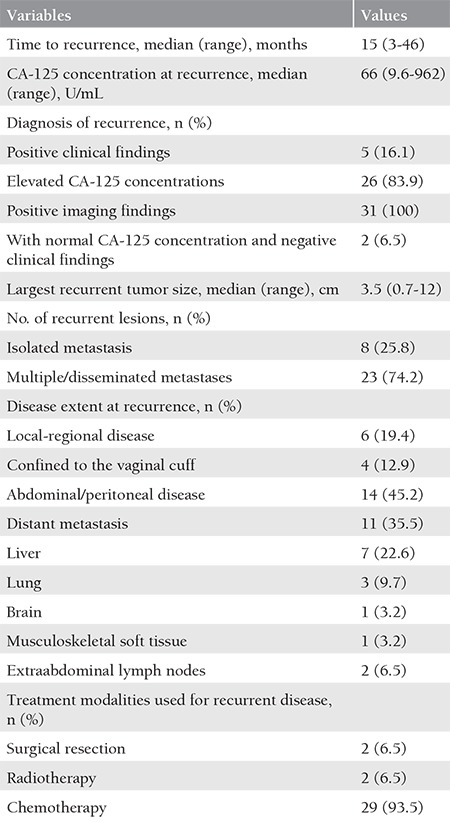
Characteristics of 31 patients with recurrent disease

**Table 3:**
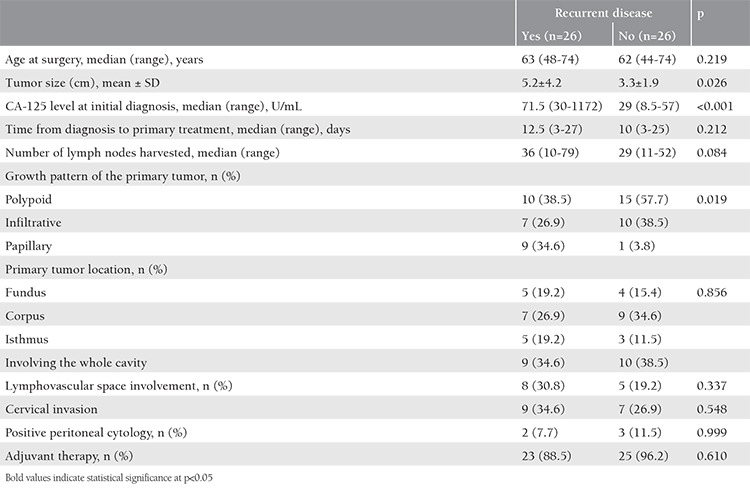
Comparison of clinicopathologic features of matched cohorts with and without recurrence

**Table 4:**
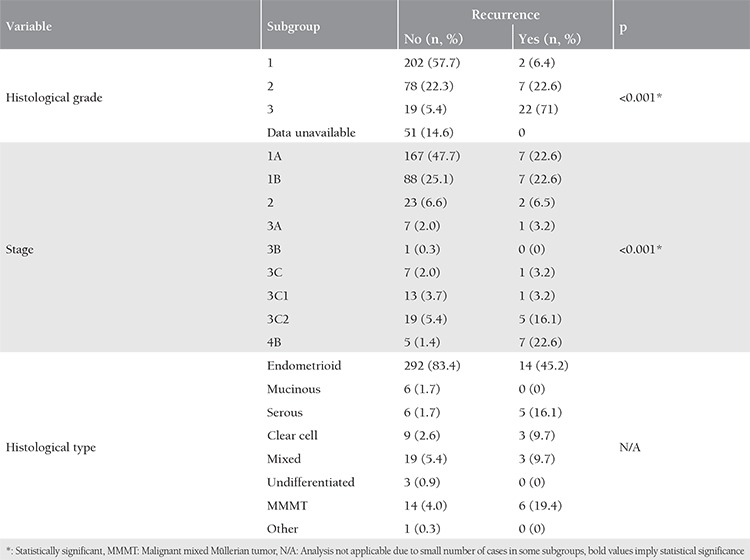
Comparison of histologic grade and type, and staging in patients with and without tumor recurrence

**Table 5:**
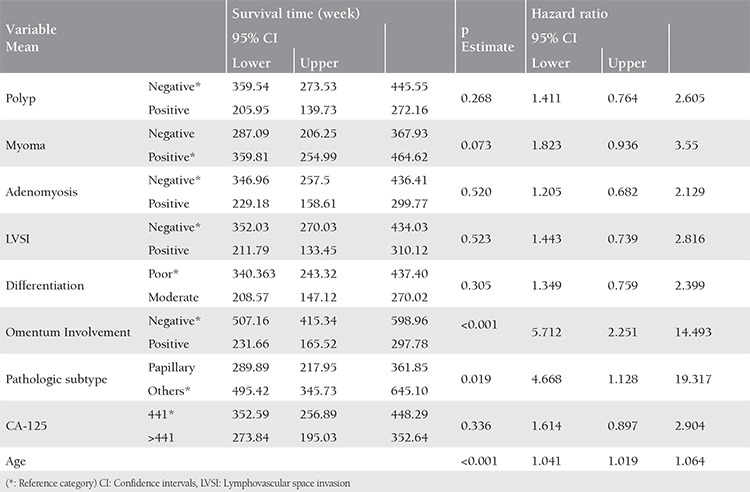
The effect of various parameters on survival
